# Human Cytomegalovirus Gene Expression in Long-Term Infected Glioma Stem Cells

**DOI:** 10.1371/journal.pone.0116178

**Published:** 2014-12-30

**Authors:** Estefania Fiallos, Jonathon Judkins, Lisa Matlaf, Mark Prichard, Dirk Dittmer, Charles Cobbs, Liliana Soroceanu

**Affiliations:** 1 California Pacific Medical Center Research Institute, San Francisco, California, United States of America; 2 Department of Pediatrics and Infectious Disease, University of Alabama at Birmingham, Birmingham, Alabama, United States of America; 3 Department of Virology, University of North Carolina School of Medicine, Chapel Hill, North Carolina, United States of America; 4 The Ben and Catherine Ivy Center for Advanced Brain Tumor Treatment, Swedish Neuroscience Institute, Seattle, Washington, United States of America; 5 Department of Neurosurgery, University of California San Francisco, San Francisco, California, United States of America; University of Regensburg, Germany

## Abstract

The most common adult primary brain tumor, glioblastoma (GBM), is characterized by fifteen months median patient survival and has no clear etiology. We and others have identified the presence of human cytomegalovirus (HCMV) gene products endogenously expressed in GBM tissue and primary cells, with a subset of viral genes being consistently expressed in most samples. Among these viral genes, several have important oncomodulatory properties, regulating tumor stemness, proliferation, immune evasion, invasion and angiogenesis. These findings lead us to hypothesize that a specific HCMV gene signature may be associated with GBM pathogenesis. To investigate this hypothesis, we used glioma cell lines and primary glioma stem-like cells (GSC) infected with clinical and laboratory HCMV strains and measured relative viral gene expression levels along several time points up to 15 weeks post-infection. While HCMV gene expression was detected in several infected glioma lines through week 5 post-infection, only HCMV-infected GSC expressed viral gene products 15 weeks post-infection. Efficiency of infection across time was higher in GSC compared to cell lines. Importantly, HCMV-infected GSC outlived their uninfected counterparts, and this extended survival was paralleled by increased tumorsphere frequency and upregulation of stemness regulators, such as SOX2, p-STAT3, and BMX (a novel HCMV target identified in this study). Interleukin 6 (IL-6) treatment significantly upregulated HCMV gene expression in long-term infected glioma cultures, suggesting that pro-inflammatory signaling in the tumor milieu may further augment HCMV gene expression and subsequent tumor progression driven by viral-induced cellular signaling. Together, our data support a critical role for long-term, low-level HCMV infection in promoting survival, stemness, and proliferation of GSC that could significantly contribute to GBM pathogenesis.

## Introduction

Glioblastoma multiforme (GBM), a grade IV glioma, is the most aggressive and malignant type of brain tumor [1]. The cause of GBM remains unknown, and even with current treatments, the median survival for patients with GBM is 15 months [2]. Glioma stem-like cells (GSC) constitute a small subset of tumor cells characterized by expression of various stem cell markers and endowed with tumor initiating capabilities (reviewed in [3]). GSC are resistant to radiation and chemotherapy and are primarily responsible for GBM recurrence [4].

There is an increased interest in elucidating the role of human cytomegalovirus (HCMV) in cancer since it has been associated with GBM and several other malignancies (reviewed in [5]). Our laboratory was the first to report that HCMV is present in over 95% of malignant gliomas [6], and since then, several other groups have corroborated these findings [7]–[14]. While the exact role of HCMV in GBM is still under investigation, evidence from several studies suggests that it might act as an oncomodulator, altering proliferative signaling, cell growth, angiogenesis, cell death, immune detection, and chromosome stability (reviewed in [15]).

HCMV is a herpes virus affecting 50–80% of the population. HCMV infection can persist for the lifetime of the host in adult stem cells, particularly hematopoietic stem cells in the bone marrow (reviewed in [16]). There are two types of persistent viral infections: (1) latent, where no new virus is produced and (2) chronic, where new virus is produced at low levels [17]. Since HCMV gene expression pattern in endogenously infected GBM tissues is reminiscent of a low-level chronic HCMV infection (rather than a productive, lytic infection; [9], [10] and reviewed in [10], [15]) we sought to quantitatively evaluate long-term viral gene expression in HCMV-infected glioma cells using a highly sensitive RT-PCR array for detection of viral transcripts. We used T98G and U87 glioma cell lines and the primary-derived glioma stem-like (GSC) 387 and 3832 cells infected with laboratory (AD169) and clinical (TR) HCMV strains and measured viral gene expression levels for up to 15 weeks post-infection. While all cells were able to maintain HCMV infection with both virus strains for up to 5 weeks, we found that AD169-infected GSC 387 grown as neurospheres maintained detectable HCMV transcripts for the longest period of time (up to 15 weeks). We provide a comparison of viral gene expression across samples and accompanying changes in cellular gene expression, which suggest that HCMV promotes critical pathways supporting proliferation and self-renewal of GSC.

## Materials and Methods

### Ethics statement

All human brain tissues (including glioblastoma samples processed as described below) used in these studies were obtained from the CPMC Neurosurgery Department, under an IRB approved protocol (Protocol #25.125-1). All patients provided written consent stating that they allowed for their tumor samples to be used for basic research. The California Pacific Medical Center Institutional Review Board Panel #1 approved the tissue collection protocol, including the patient consent forms (Current IRB Assurance NO: FWA00000921). Samples have been de-identified before being processed, to protect patient privacy.

### Cell Lines and Cell Culture

U87 and T98G human glioblastoma cell lines were obtained from ATTC and grown in DMEM/H21 with 10% FBS. 387 and 3832 GSC lines were donated by Dr. Jeremy Rich (Cleveland Clinic) and grown as neurospheres in serum-free DMEM/F12 supplemented with N2, 20 ng/ml EGF, and 20 ng/ml FGF. Media for all cells contained 100 U/ml penicillin and 100 mcg/ml streptomycin.

### Viruses and Virus Infection

Cells were infected with either the laboratory strain AD169 (from ATCC) or the clinical strain TR (donated by Dr. Lee Fortunato, University of Idaho). Cells were incubated for 3 hours with the virus at a multiplicity of infection (MOI) of 2. Virus-containing supernatant was then replaced with fresh medium. Virus grown in serum-free medium was used for 387 cells.

### Cell Culture and IL-6 Treatment

Infected and uninfected cells were treated in the same manner throughout the experiment. To analyze cells at several time points post-infection and prevent overgrowth, T98G and U87 cells were trypsinized and re-seeded onto 6-well plates at 5×10^4^ cells/well every 3 or 4 days. 387 neurospheres were gently dissociated and expanded in 6-well plates in a similar timeframe. All cells were collected for RNA and protein extraction at 72 hrs p.i., 3 weeks p.i., 5 weeks p.i., and 7 weeks p.i. AD169-infected 387 cells were collected every two weeks up to 17 weeks p.i. To assess the effects of interleukin-6, recombinant IL-6 (R&D Systems) was added to cell medium at 50 ng/ml for 24 hrs before RNA and protein extraction at several time points.

### Genomic DNA quantification

DNA was extracted using a kit from Qiagen according to the manufacturer’s instructions. Plasmid encoding for AD169 viral DNA was used to generate a standard curve for genomic Taqman.

### RNA and Protein Isolation

Cells were washed with PBS, and total RNA was extracted using QIAshredder columns and the RNeasy Mini kit with on-column DNase digestion (Qiagen). The purity and quality of RNA was assessed by spectrometry and rRNA visualization on an agarose gel. For RT-PCR reactions, 1.5 ug RNA was reverse-transcribed in a 20-µl reaction volume using the iScript Advanced cDNA synthesis Kit (Biorad). Remaining RNA was stored at −80°C until further use. For protein extraction, cells were rinsed once with PBS and covered with RIPA buffer containing Halt Protease and Phosphatase Inhibitor Cocktail (Pierce) for 20 minutes at 4°C. Cells were collected and lysed by sonication. Whole cell lysates were centrifuged, and the supernatant was collected as total protein. Protein samples were stored at −20°C until further use.

### TaqMan RT-PCR

CMV primers and probes used for TaqMan reactions were designed by Dr. Dirk Dittmer (S1 Table). Primer/probe mixes for cellular genes (RAB14, CD44, CEPBPB, OLIG2, and SOX2) were obtained from Applied Biosystems. cDNA samples were diluted 1∶2 in nuclease free water, and a master mix using TaqMan FAST Universal PCR Master Mix (Applied Biosystems) was prepared according to the manufacturer’s instructions. Using a final reaction volume of 11 ul, RT-PCR reactions were performed and analyzed with the ViiA7 Real-Time PCR System (Applied Biosystems).

### SYBR Green RT-PCR

To run high throughput RT-PCR reactions, we used 384-well plates, a 7 ul reaction volume, and pre-made plates with 3 uM CMV primers following high-throughput RT-PCR methods [18]. A master mix containing cDNA, nuclease-free water, and Fast SYBR Green master mix was prepared to run each array, and primers were added with a multichannel pipette. In the final PCR reaction, each well contained 0.039 ul cDNA, 1.361 ul nuclease-free water, 2.1 ul primer mix (forward and reverse primers at 3 uM each), and 3.5 ul of 2X Fast SYBR Green master mix (Applied Biosystems). The reaction was performed using the Vii7A Real-Time PCR System (Applied Biosystems). Melting curves were generated to confirm amplification specificity of each PCR product. ΔCts for viral genes were calculated by normalizing to the housekeeping gene RPL13A, and relative fold changes in gene expression were calculated using the 2^−ΔC^_T_ formula [19].

### Primer design and validation for SYBR Green RT-PCR

All primers used in this study for SYBR Green RT-PCR are shown in S2 Table. The performance of the primers was initially validated in infected cells and compared to uninfected cells to confirm the specificity of the primers and identify the melting temperature of the products. All of the primers were also evaluated in the absence of reverse transcriptase to ensure that the signal observed was from RNA rather than contaminating viral DNA. Finally, RNA was isolated from a time course experiment to show that the viral mRNA increased with time according to the expected kinetics. Since the primers were designed for the AD169 virus strain, we aligned all primers against the TR viral genome and eliminated any primers with mismatches from further analysis in TR-infected samples. To obtain specific melting points for each viral gene, viral genes were amplified from AD169 DNA and U87 cells infected with TR at high MOI. Any amplification product with a melting point that did not match this specific melting point within 1°C was considered a non-specific product. Since there might be non-specific products with a melting point close to the viral gene melting point, uninfected samples of all cells (U87, T98G, and 387) were run along all viral gene primers. Any primers producing non-specific amplification with a Tm close to the expected viral gene Tm were eliminated from analysis in those cell samples. For TR-infected samples, a total of 55 genes were tested in U87 cells, 57 in T98G cells, and 54 genes in 387 GSC. In the AD169 infection, a total of 94, 108, and 85 genes were tested in U87, T98G, and 387 cells, respectively.

### Tumorsphere Limited Dilution Assays

HCMV (TR, MOI = 2) infected GSC (387 and 3832) were sorted for CD44 and CD133 respectively, as described in reference [20]. For limited dilution assays, cells were plated (after sorting) at 10, 100, 1000 cells/well in 24 wells (6 wells/condition). Tumorsphere formation was monitored using an inverted Nikon microscope fitted with a camera. The number of wells with spheres was recorded on day 10 after culturing. The experiment was repeated twice. Data analysis performed using the publically available ELDA software as detailed in reference [21].

### Immunofluorescence

Immunofluorescence and quantification of infected glioma and GSC cultures was performed as previously published by our group [22]. Culturing GSC on laminin coated surfaces (rather than in spheres) has been shown to maintain their undifferentiated status and not alter their genome for up to 60 weeks [3]; therefore, cell samples were placed on laminin coated coverslips. Cells were probed with anti-IE1 (mAB810, Millipore), anti-Sox2 (Abcam), and anti-Nestin (Abcam) primary antibodies, then with AlexaFluor488 anti-mouse secondary antibody. Nuclei were counterstained with DAPI. IE1+ nuclei were counted with a fluorescence microscope fitted with a camera. Efficiency of infection was defined as the number of IE1+ cells divided by the total number of cells times 100.

### Western Blot

Standard western blot procedures were employed. Primary antibodies include anti-IE1 (mAB810, Millipore), anti-actin (Sigma), anti-phospho-STAT3 (#9145, Cell Signaling), anti-STAT3 (#9132, Cell Signaling), anti-BMX (ab32153, Abcam). Secondary antibodies used were goat anti-rabbit HRP and goat anti-mouse HRP (31460 and 31430, Thermo Scientific). Protein bands were developed on a film with SuperSignal Chemiluminescent Substrate (Thermo Scientific) and analyzed by densitometry in Image J. Target proteins were normalized to actin.

### Statistical analyses

Grouped data are shown as mean ± SD. Where appropriate, student t-test and ANOVA were used to establish significant differences.

## Results

### HCMV gene products are detectable five weeks post-infection in several GBM lines

**Figure 1:**
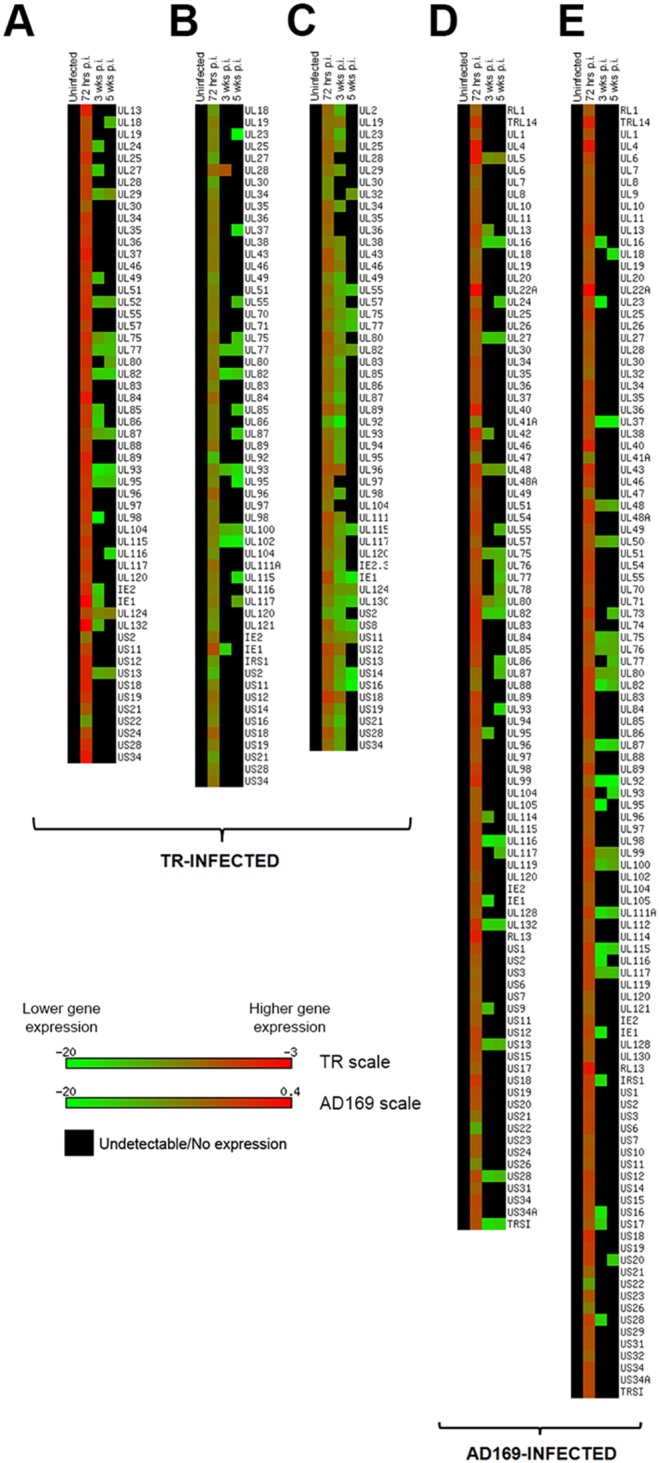
Heatmap representation of relative abundance of HCMV gene expression in infected glioma cells. All cells were infected with a strain of HCMV (MOI = 2). RNA was extracted at 72 hours p.i., 3 weeks p.i., and 5 weeks p.i. for gene expression analysis via SYBR green RT-PCR. Note that not all genes could be tested on each sample. The length of the heatmap represents the total number of genes tested for that particular sample. Ct values were normalized with housekeeping gene RPL13A to obtain ΔCt values, which were inputted into the Matrix2png program available at http://chibi.ubc.ca/matrix2png/index.html to generate the heatmap representation of relative transcript abundance. Green shows lower viral gene expression and red depicts higher expression. Black indicates that either the gene is not expressed or is below the limit of detection. A. U87 cells infected with TR virus. B. T98G cells infected with TR virus. C. 387 cells infected with TR virus. D. U87 cells infected with AD169 virus. E. T98G cells infected with AD169 virus.

**Figure 2:**
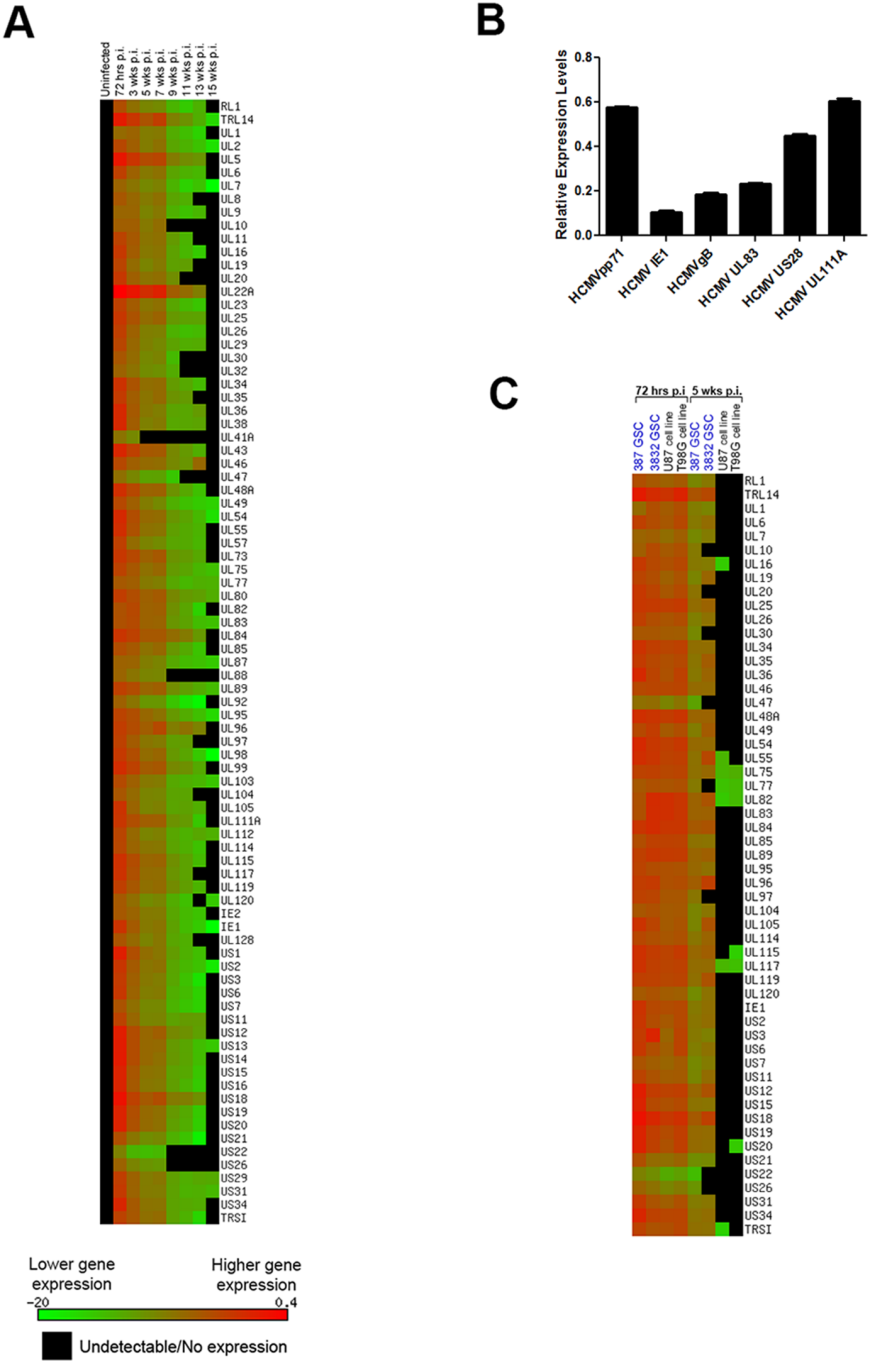
HCMV gene expression is detectable at 15 weeks p.i only in 387 GSC cells. A. 387 GSC cells infected with AD169 virus (MOI = 2) were analyzed for viral gene expression with SYBR Green RT-PCR at 72 hours, 3 weeks and every 2 weeks thereafter up to 15 weeks. B. Taqman validation of a subset of viral genes of AD169-infected 387 cells at 11 weeks p.i. Relative expression levels were obtained by normalizing viral gene expression with expression of cellular gene Rab14. Each sample was run in triplicate, bars represent S.D. C. Comparison of viral gene expression of AD169-infected glioma stem-like cells and cell lines at 72 hours and 5 weeks p.i. 3832 GSC cells were infected with AD169 virus (MOI = 2) and analyzed for viral gene expression with SYBR Green RT-PCR at 72 hours and 5 weeks p.i. These results are displayed alongside 72 hr and 5 week p.i. data for AD169-infected 387, U87, and T98G from Figures 1D–E and Figure 2A.

In order to assess long-term HCMV infection of glioblastoma cells, we infected two established glioblastoma cell lines, T98G and U87, as well as the primary 387 GSC with two HCMV strains–the clinical strain TR and the laboratory strain AD169. Cells were continuously maintained in culture and tested for HCMV gene expression every two weeks, until CMV RNA was no longer detectable or cellular senescence ensued. To monitor global gene expression at different time points post infection, we used a SYBR Green-based RT-PCR array with custom-made CMV primers and used melting curve analyses to check the specificity of the amplified product. Due to primer optimization for each cell and virus strain, a different number of genes was tested for each sample (Figs. 1 and 2A). The RT-PCR array was originally designed for the AD169 strain, so any primers that did not align with the TR genome were eliminated from TR-virus runs. As a result, about twice as many genes were tested in AD169-infected cells than in those infected with TR. Furthermore, since SYBR green dye amplifies any double stranded DNA, we tested uninfected cells for any non-specific PCR amplification products with melting points that could be mistaken for the target gene melting point and excluded these primer sets from analyses. After these optimization steps, the total number of genes tested in the TR infection in U87 cells was 55, 57 genes in T98G, and 54 genes in 387 GSC (Fig. 1A–C). In the AD169 infection, 94 genes were tested in U87 cells, 108 in T98G, and 85 genes in 387 GSC (Fig. 1D–E and Fig. 2A). To further verify that the primers were amplifying the correct viral genes, we sequenced several PCR products from AD169 and TR-infected T98G cells 72 hrs post-infection (p.i.). The sequences obtained correctly aligned with the corresponding gene in the HCMV AD169 or TR genome (S1 Fig.).

Viral gene transcripts were detected up to five weeks p.i. in TR-infected U87, T98G, and 387 cells, as well as AD169-infected U87 and T98G cells (Fig. 1A–E). In U87 and T98G cells infected with TR or AD169, viral expression decreased sharply at three weeks p.i. A larger proportion of viral genes was detectable at three weeks p.i. in TR-infected 387 GSC (87% of genes tested) compared with U87 and T98G cells infected either with TR, where 35% and 12% of genes tested were detected at three weeks, respectively, or with AD169, where 22% of genes were detected in U87 and 18% were detected in T98G cells (Fig. 1 A–C).

### HCMV transcripts are detected in AD169-infected 387 GSC for up to fifteen weeks post-infection

In primary-derived 387 GSC infected with AD169 virus, viral transcripts were detectable up to 15 weeks p.i. (Fig. 2A). To confirm the presence of CMV transcripts at late time points, an infected sample at 11 weeks p.i. was quantified by TaqMan for the following gene products: IE1 (UL123), US28, UL55 (gB), UL83 (pp65), and UL111A (Fig. 2B).

To test whether the difference in the length of infection could be attributed to characteristics specific to glioma stem-like cells, we analyzed an additional type of primary-derived glioma stem-like cells: 3832 GSC. 3832 GSC were infected with AD169 following the same protocol, and gene expression was analyzed at 72 hours and 5 weeks p.i. Fig. 2C shows a comparison of the relative abundance of viral genes in GSC (387 and 3832) versus cell lines (U87 and T98G) at 72 hrs and 5 weeks after AD169 infection. Only genes tested in all four samples were chosen for this comparison (n = 56). While all samples show high levels of infection at 72 hrs p.i., there is a clear difference in transcript levels at 5 weeks p.i., where 100% and 85% of the 56 genes are still expressed in 387 and 3832 GSC, respectively, while only 11% of genes remain present in U87 and T98G cell lines at very low levels of expression. Note that 3832 GSC were only used for this comparison and were not analyzed past 5 weeks p.i.

### HCMV efficiency of infection is superior in GSC compared to standard glioma cell lines

**Figure 3:**
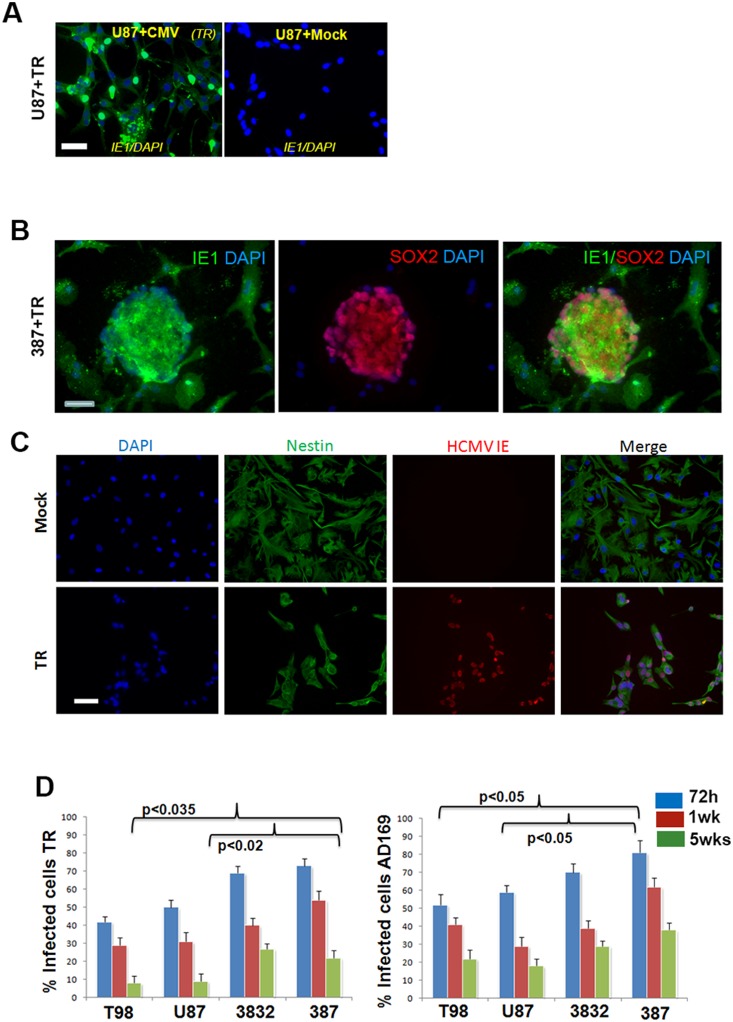
HCMV Infection Efficiency. A. U87 cells cultured on cover glass and treated with mock or TR (MOI = 2) were fixed with ethanol (72 hrs p.i.) and processed for IE1 immunofluorescence in conjunction with an Alexa 488-labeled secondary antibody. Nuclei were counterstained with DAPI. Bar = 50 um. B. GSC 387 grown as neurospheres were infected with TR (MOI = 2) and stained for IE1 and Sox2. Photomicrographs demonstrate co-localization of the two markers. Bar = 100 um. C. To quantitatively assess HCMV infection efficiency in GSC, 387 cells were cultured on laminin, treated with mock or TR (MOI = 2) and 72 h later processed for IE1 and Nestin immunofluorescence. Nestin is a marker of un-differentiation abundant in GSC. Nuclei were counterstained with DAPI. Rightmost panels show superimposed images of Nestin+, IE1+ cells. Bar = 100 um. D. Cell lines and GSC cultures treated as described above were counted using a fluorescence microscope fitted with a camera. Four low magnification fields were counted for each condition (repeated in duplicate coverslips). Percentage of positive cells was calculated by dividing the number of IE1+ cells to the total number of nuclei and multiplying by 100. Student t-test was used to compare the number of 387 IE1 positive cells at 5 weeks p.i. (green bars) with the number of T98G and U87 IE1 positive cells, respectively. For both viral strains, differences between IE1+ cells in GSC and cell lines were significant (p values displayed).

To evaluate the impact of infection efficiency between two viral strains across four cell lines, we infected U87, T98G, 387 and 3832 cells with AD169 or TR (MOI = 2) and maintained the cells in culture without re-infection for five weeks. Cells were placed on laminin coated coverslips to allow for immunofluorescent detection of IE1 and quantification of CMV positive cells. Culturing GSC on laminin coated surfaces (rather than in spheres) has been shown to maintain their undifferentiated status and not alter their genome for up to 60 weeks [3]. Fig. 3 shows photomicrographs obtained from U87 and 387 cells infected with TR and processed for IE1 immunofluorescence. 72 hours post infection GSC 387 and 3832 were more efficiently infected compared to cell lines T98G and U87 (70–80% IE1 positive as compared to ∼40%). By week five, the percentage of IE1 positive cells was ∼40% in 387 cells as compared to 20% in U87 (for AD169), suggesting that GSC maintain HCMV infection more effectively than standard, serum-maintained glioma cell lines.

### HCMV viral DNA is maintained at higher levels in primary GSC as compared to glioma cell lines

We performed Taqman for HCMV genes US28, pp65 and pp71 using genomic DNA isolated from GSC 387 and T98G infected with AD169 (MOI = 2) up to five weeks. Results from 72 h, 2 weeks, 3 weeks and 5 weeks show that viral DNA maintained relatively higher levels in GSC 387 as compared to T98G, which is in agreement with measurements of viral transcripts in the same lines (S2 Fig.).

### HCMV protein products are detected at late time points

**Figure 4:**
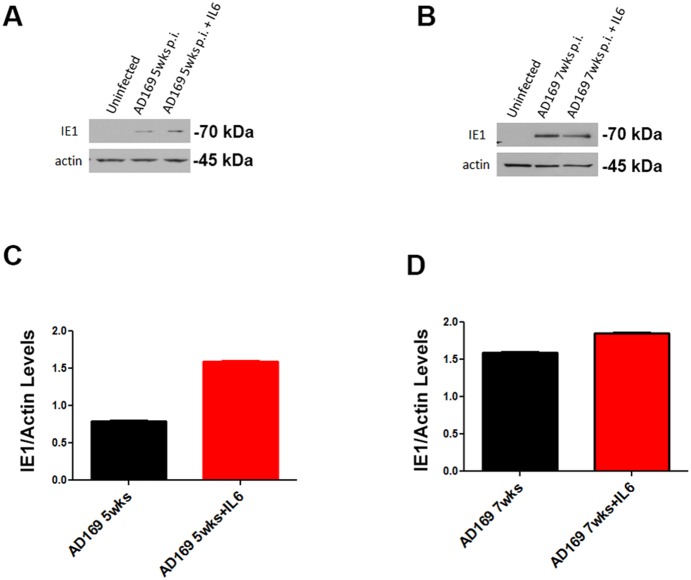
HCMV IE1 protein is expressed in AD169-infected 387 cells at late time points p.i. A and B. 387 GSC cells treated with vehicle or IL-6 before protein collection (5 weeks and 7 weeks p.i) were subjected to western blot analysis for the presence of HCMV IE1 protein. Actin was used as a loading control. C and D. Densitometric analyses of western blots shown in A and B, respectively. Pixel density of IE1 bands was normalized with actin bands to obtain relative IE1 levels. Note: A and B represent different exposure times; therefore, protein levels cannot be compared between 5-week and 7-week samples.

To further validate the presence of the viral gene products at late time points in the AD169-infected 387 sample, we used western blot analysis of samples collected 5, 7, 9, and 11 weeks p.i. (Fig. 4). IE1 is clearly detected at 5 and 7 weeks p.i. and the protein levels are augmented by IL-6 treatment (Fig. 4A–D). By contrast, we could not detect IE1 protein in TR-infected 387 GSC three or five weeks p.i. (not shown). IE1 protein appears to be weakly expressed at 9 and 11 weeks p.i. in the AD169-infected GSC 387 (S3 Fig.).

### Interleukin-6 upregulates CMV gene expression in GBM lines

**Figure 5:**
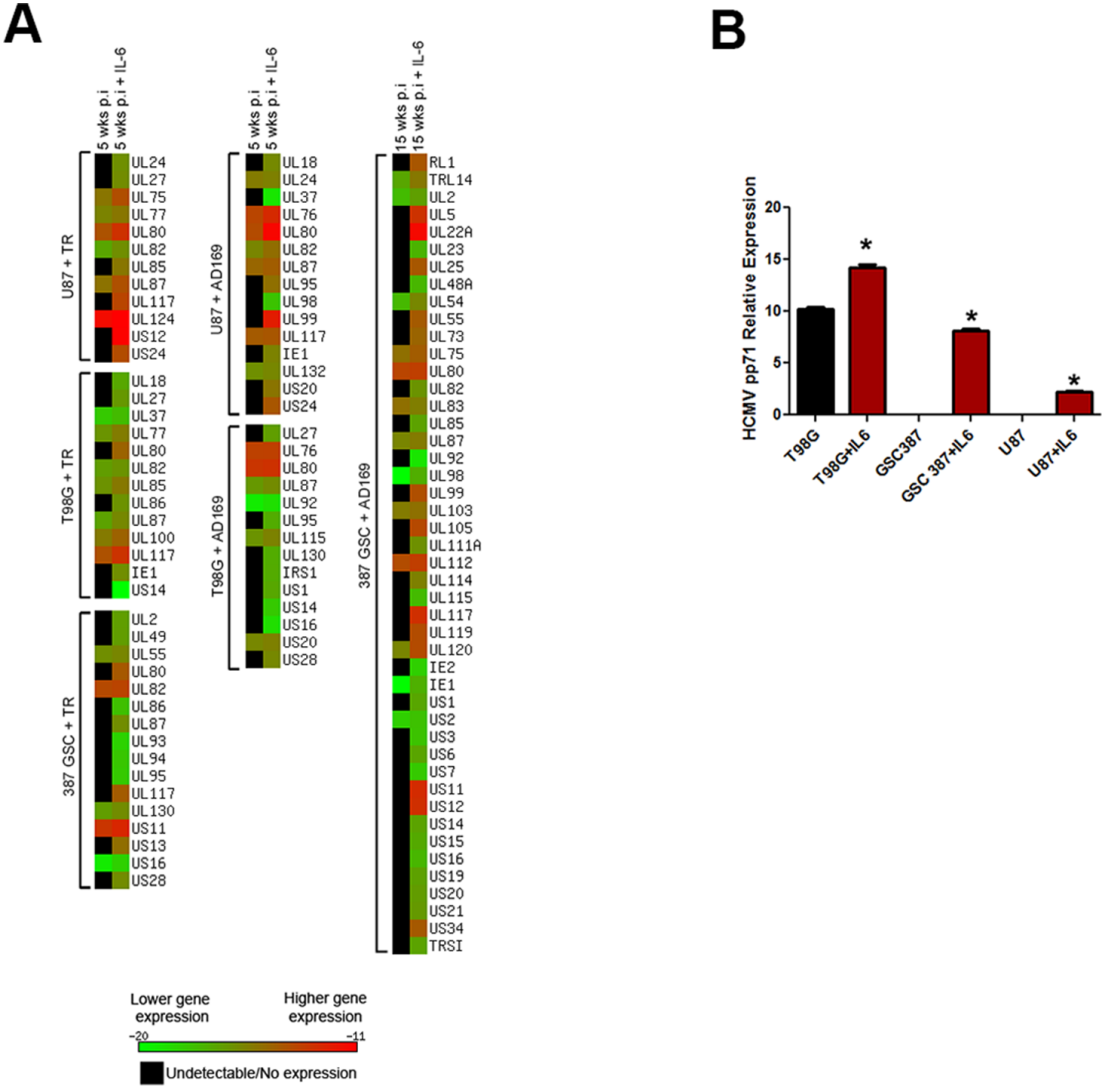
IL-6 treatment upregulates viral gene expression in HCMV-infected GBM cells. At 5 weeks p.i. (or at 15 wks p.i. for AD169-infected 387 GSC) a subset of cells was treated with IL-6 for 24 hours before RNA extraction. A. Heatmap representation of viral gene expression in in TR or AD169-infected U87, T98G, and 387 GSC with or without IL-6 treatment B. HCMV pp71 was detected using Taqman in TR-infected T98G, TR-infected 387, and AD169-infected U87 cells. Mean values normalized to Rab14 are shown. Each sample was run in quadruplicate. *p<0.02 student T-test.

We used IL-6 treatment at late time points post-infection to induce reactivation of HCMV gene expression in long-term infected glioma cells. While several HCMV transcripts were upregulated by IL-6 treatment across all samples, only AD169-infected 387 GSC demonstrated upregulation of a subset of HCMV genes as late as 15 weeks p.i., in response to IL-6 (Fig. 5A). To validate the array results, we used TaqMan analysis and were able to measure significant upregulation of HCMV pp71 at 5 weeks p.i. in three samples (TR-infected T98G, TR-infected 387 GSC, and AD169-infected U87) treated with IL-6 compared to controls (Fig. 5B).

### HCMV infection upregulates stemness regulators in GSC

**Figure 6:**
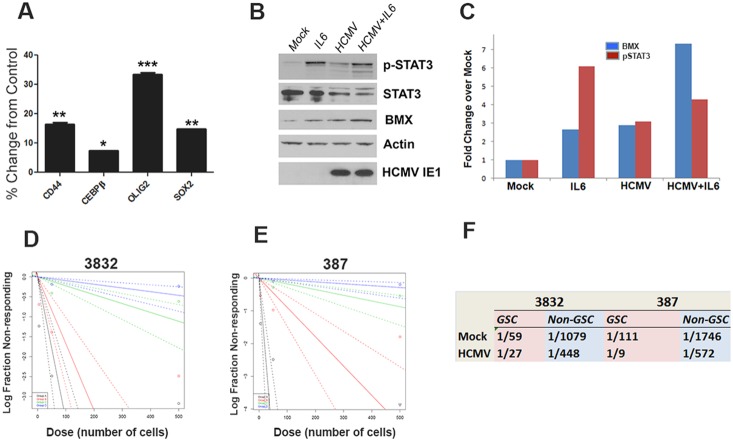
HCMV induces BMX-p-STAT3 pathway in GSC 387. A. RNA was collected from AD169-infected and uninfected 387 cells at 5 weeks p.i for gene expression analysis of four cellular genes. Mean values are shown as the percent increase in gene expression in AD169-infected samples compared to uninfected cells. Each sample was run in triplicate. Paired t-test *p<0.02, **p<0.05, ***p<0.005. B. HCMV AD169 and mock-infected 387 cells were treated with IL-6 for 24 hours before protein extraction (5 weeks p.i.). Phospho-STAT3, total STAT3, BMX, HCMV IE1 and actin were detected using western blot analyses with the indicated antibodies. C. Densitometric analysis of BMX and p-STAT3. Protein expression was normalized to actin and is expressed relative to the mock-infected sample (assigned a value of 1). D. Limited dilution assays performed with 3832 GSC sorted for CD133 and mock or HCMV-infected (TR, MOI = 2). 10, 100, 1000 cells/well in 24 well plates were assayed. Wells with spheres were counted at 10 days post initial culturing. Results were analyzed and plotted using freely available software for predicting cancer stem cell frequency http://bioinf.wehi.edu.au/software/elda/. E. Same experiment as described in D was run using the CD44 positive and negative fractions of GSC 387 line treated with mock or HCMV (TR, MOI = 2). F. Estimated glioma stem cell frequency for the indicated conditions. Overall test for differences in stem cell frequencies between any of the groups: p = 1.68e-5 for GSC 387 and p = 0.0083 for GSC 3832.

Mock-infected 387 GSC were maintained in culture in parallel with AD169-infected GSC; however, the uninfected cells began to senesce shortly after the 5-week time point while the infected cells continued to proliferate. Therefore, we decided to interrogate changes in cellular gene expression that could induce infected GSC to outlive their uninfected counterparts. At 5 weeks p.i., several cellular transcripts, markers of GBM stemness and aggressive (mesenchymal) signature, were upregulated in the AD169-infected 387 GSC compared to the mock-infected samples. These are CD44, CEBPβ, OLIG2, and SOX2 (Fig. 6A). Interestingly, we also found upregulation of BMX levels and activation of its downstream target, phospho-STAT3, in the HCMV-infected GSC (Fig. 6B–C). BMX is a regulator of glioblastoma stemness, via activation of the p-STAT3 pathway [23]. IL-6 treatment used to model the pro-inflammatory environment of a HCMV-positive GBM further augmented the BMX-p-STAT3 pathway in both infected and uninfected GSC (Fig. 6B–C).

3832 GSC line, which expresses CD133 was HCMV infected (TR, MOI = 2) and sorted in stem and non-stem like fraction using this well-described GSC marker. Limited dilution assays showed that HCMV infection increased the frequency of tumorspheres in both CD133 positive and negative 3832 fractions (Fig. 6D, F). HCMV and mock-infected 387 GSC were sorted for CD44 (a mesenchymal stemness marker) and subjected to limited dilution tumorsphere assays. Our data shows that the stem cell frequency was highest in the HCMV+ CD44+ cells (Fig. 6E–F). Interestingly, profiling of HCMV-infected 3832 GSC shows that the CD133+ (stem-like) cell fraction is enriched in viral transcripts, as compared to the CD133 negative fraction (S4 Fig.).

### A subset of HCMV gene products are expressed across all glioma samples at late time points post infection

**Figure 7:**
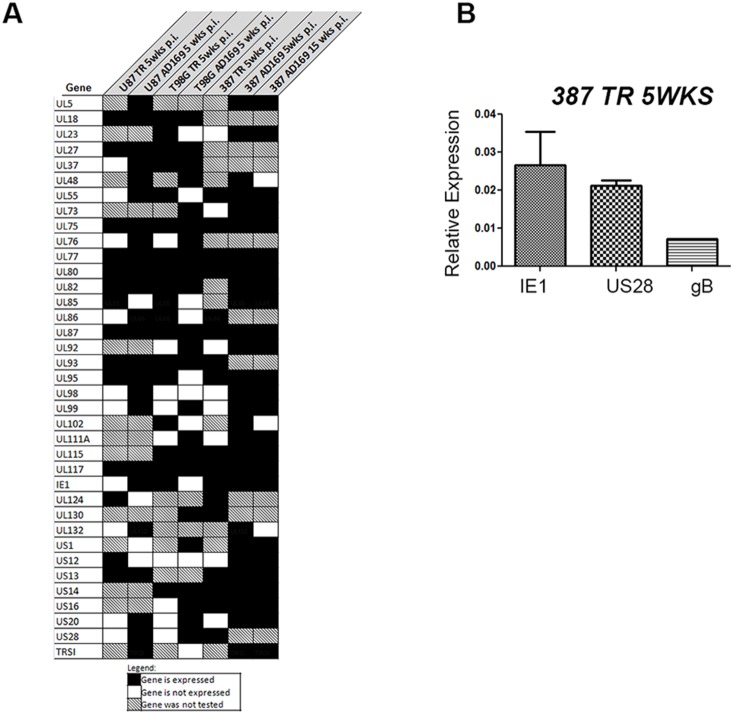
Graphic representation of HCMV gene expression at late time points in all cell lines and viral strains tested. A. Viral genes with detectable expression at 5 wks p.i. and 5 wks p.i. + IL-6 (also 15 wks p.i. and 15 wks p.i. + IL-6 for AD169-infected 387 cells) across U87, T98G, and 387 GSC are shown. A square with striped background indicates that the gene was not tested for that particular sample. B. Taqman validation for IE1, US28, and gB gene expression in GSC 387 infected with TR (5 weeks p.i). Mean values normalized to Rab14 are shown. Samples were run in triplicate. Error bars are displayed.

We assessed viral transcripts expressed at late time points across all GBM lines and viral strains, including samples collected at 5 weeks and 15 weeks p.i, as well as IL-6-treated samples at those time points. We identified a subset of 37 viral genes that were expressed in at least two different GBM lines at late time points and several genes, including UL75, UL77, UL80, UL87, and UL117, which were expressed across all cells and viruses (Fig. 7A). TaqMan validation for 387 GSC TR-infected sample confirmed expression of IE1, UL55 (gB) and US28 at 5 weeks p.i. (Fig. 7B).

## Discussion

### Glioma stem-like cells as a model for HCMV chronic infection in GBM

HCMV gene expression in endogenously infected glioblastoma does not fit the classic definition of latency (i.e. the maintenance of viral genomes without lytic gene expression or virus production) since most GBM samples described to date exhibit expression of IE1 (UL123), a lytic gene [6]–[10], [13]. A more suitable definition of HCMV infection in glioblastoma has been postulated as a chronic infection with viral gene expression but no cytopathic effect [15]. Our study aimed to assess patterns of long-term HCMV infection in glioblastoma cell lines and primary GSC cultures, which could serve as a model for studying the effects of HCMV endogenous infection in GBM pathogenesis. We infected cells with the laboratory strain AD169 since a previous study demonstrated that it could maintain long-term infection in the T98G glioblastoma cell line [24], and the TR strain was used for comparison with a clinical strain. Our study confirms the persistence of HCMV transcripts in the T98G cell line and shows that U87 and the primary-derived 387 GSC may also be used as models for chronic HCMV infection. In particular, AD169-infected 387 GSC show remarkably long maintenance of HCMV transcript expression (15 weeks p.i.).

Using IE1 immunofluorescence, we also determined that efficiency of infection was higher in GSC lines compared with standard glioma cell lines at late times post-infection. However, reduced gene expression in T98G and U87 cells at late time points cannot be fully attributed to reduction in the efficiency of infection since the decrease in transcript levels of cell lines relative to GSC at 5 weeks is greater than the difference in the number of IE1 positive cells. The enhanced maintenance of infection in GSC is most likely related to the stem-like properties of these cells.

We did not observe the same maintenance of HCMV expression in 387 GSC cells infected with the TR strain. This difference might arise because, as a lab-adapted strain, AD169 is better suited to be maintained in cultured cells. Also, since HCMV is a highly polymorphic virus, this difference could reflect differential virus-host interaction between TR and AD169 strains. For example, there appear to be variations in the protein complex that mediates viral entry into the host between TR and AD169 strains that can lead to differences in infectivity [25], [26].

While there was a general trend of decreasing gene expression over time across samples, several genes that were not present at 3 weeks p.i. were detected at 5 weeks p.i (e.g. UL18 in Fig. 1A). This could be due to different patterns of expression across the life cycle of the virus. It is possible that at certain time points, the level of expression drops below the level of detection and later reappears with increased transcription of a particular gene in response to signaling changes in the virus. Also, at very low levels of the virus, detection of the transcript becomes a rare event with low probability of detection. We performed at least 6 replicates to detect gene expression in samples with low viral levels to prevent false negatives, but it is still possible that the virus at 3 weeks p.i. was missed and that it might be detected by performing more replicates of the reaction.

### HCMV promotes GSC stemness and self-renewal

It has been proposed that the reason GBM evades therapy is the persistence of a small population of glioblastoma stem cells that hold the potential to self-renew, differentiate into multiple lineages, and initiate tumors (reviewed in [3]). Primary tumor-derived cells grown in neurospheres like the 387 and 3832 cells used in this study are enriched in stem-like cells. Two of the markers used to identify stem-like cells are CD133 and CD44 [27], [28]. We have previously observed that HCMV gene products are expressed at higher levels in CD133+ stem-like cell fractions (S4 Fig. and [22]). HCMV infection significantly increased stem cell frequency in the CD44+387 cells as well as in the CD133+ fraction of 3832 cells. We recognize that GSC markers, including CD144 and CD44, are not sufficient to functionally characterize a cancer stem cell, therefore additional studies are needed to determine the role of HCMV in GSC biology *in vivo*. Our mechanistic analyses demonstrate that HCMV coordinately upregulated the BMX-p-STAT3 pathway and, notably, HCMV infected 387 GSC outlived by several weeks their uninfected counterparts. In addition, several markers of stemness and aggressive phenotype, including SOX2, OLIG2, and C-BEPβ were upregulated in long-term infected 387 GSC compared uninfected cells. Taken together, these data argue for a role of HCMV in the maintenance of GBM stemness and self-renewal. Since glioblastoma-derived sphere cultures have also been shown to be more representative of the parent tumor [29], our data presented herein suggest that GSC may serve as a suitable model to study the effects of chronic HCMV infection in GBM.

Since GSC share some characteristics with normal neural precursor cells (NPC), we further attempted to replicate the long term HCMV infection experiments in NPC, however these cells differentiated and underwent apoptosis within ∼15 days p.i., as previously reported by other groups [30]. We speculate that the differential effects HCMV exerts onto normal and cancerous neural stem cells are in part due to the differences in the differentiation state of cells (NPC vs GSC) and the presence of additional DNA mutations in GSC (but not in NPC), which regulate both viral and cellular gene expression. In addition, BMX, which is preferentially expressed in GSC over NPC, regulates activation of STAT3 and expression of SOX2 and OLIG2 in GSC but not in NPC [23].

### Interleukin-6 upregulates HCMV transcription

IL-6 can reactivate latent HCMV virus in CD14+ monocytes and dendritic cells [31], [32], and it is a pro-inflammatory cytokine commonly found in the tumor environment. Although our model does not represent a classical latent infection, we hypothesized that IL-6 would also promote HCMV transcription during a chronic infection. Our results show that treatment with IL-6 can upregulate CMV gene and protein expression. Several viral genes that were previously undetectable showed expression after IL-6 treatment. However, the effects of IL-6 were only observed for a subset of genes at late time points while upregulation was observed across the majority of genes at earlier time points. This suggests that IL-6 upregulated expression of viral genes that were expressed at low levels below the limit of detection rather than reactivating genes that had ceased transcription altogether. These data suggest that long term infected GSC represent a model of a low-level chronic infection rather than a latent HCMV infection.

### BMX as a novel target for HCMV and the HCMV-IL6-BMX tumor-promoting pathway

**Figure 8:**
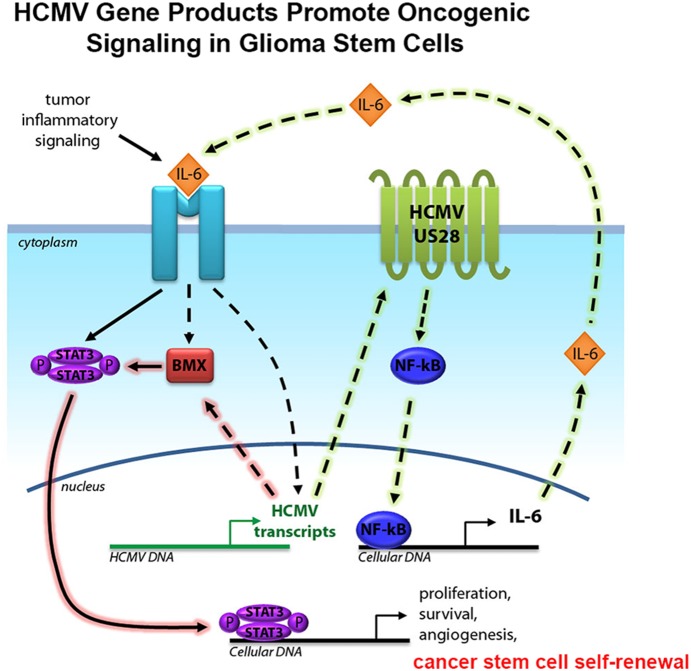
Diagram represents proposed signaling pathways between HCMV and the IL6-BMXp-STAT3 axis in long-term HCMV infected human glioma stem cells.

IL-6 treatment also upregulated BMX and pSTAT3 proteins in both infected and uninfected samples, The bone marrow X-linked kinase (BMX) is enriched in GSC compared to astrocytes and neural progenitor cells, and BMX knockdown suppresses tumor growth [23]. Both IL-6 and BMX can activate transcription factor STAT3, which drives GSC proliferation and maintenance [23], [33]. Consistent with previous studies, we show increased levels of STAT3 phosphorylation after HCMV infection [34]. BMX protein expression also increased in response to HCMV infection without IL-6 treatment; therefore, our results identify BMX, a regulator of GBM stemness via STAT3 activation, as a novel target of HCMV. The relationships between HCMV, BMX, and IL-6 suggest a positive feedback loop in a signaling pathway leading to tumor cell proliferation and maintenance. It has been shown that the viral G protein-like receptor US28, which is expressed in a high number of GBMs [35], activates the IL-6-JAK1-STAT3 signaling axis [34], [35]. It is possible that BMX may be part of the US28 signaling pathway, but further studies will be necessary to establish the exact relationship between HCMV and BMX. The diagram in Fig. 8 illustrates a putative signaling pathway activated by HCMV, leading to GBM hallmarks through STAT3 activation.

### A gene signature of HCMV chronic infection

While HCMV encodes for over 150 genes, only a subset are expressed at low levels during chronic infections. To find this potential gene signature, we looked for genes that were expressed in at least two different GBM cell lines at late time points. Our analysis is limited by optimization procedures that led to the exclusion of several genes in the analysis of each sample and the use of only one GSC line at late time points of chronic infection. A more accurate picture of the gene signature in chronic HCMV infection might be provided through analysis of several GSC lines infected with AD169 since this model might most closely represent the HCMV chronic infection in GBM. Within these limitations, we have identified a subset of 37 genes expressed at late time points of infection, and the majority of these genes have not been previously studied in the context of glioblastoma. We have previously shown how five of these genes–IE1, UL82 (pp71), UL55 (gB), and US28–contribute to oncogenic signaling by promoting cell proliferation, angiogenesis, and invasion [22], [35]–[37]. We measured the presence of one latency-associated transcript (UL111A) in GSC 387 and T98G infected with AD169 at late time points (Fig. 7). However, other classical HCMV latency transcripts, such as UL138, UL144, and LUNA, previously detected in latently infected myeloid cells, were not found in long term infected GSC. This discrepancy in latent viral gene expression is likely due to cell type specific regulation of HCMV expression and reactivation. We now show for the first time that HCMV UL75, UL77, UL80, UL87 and UL117 are expressed in both TR and AD169-chronically infected GBM cells. Glycoprotein H (UL75) is an envelope protein that mediates viral entry and activation of gene expression [38], [39], while UL77 and UL80 are essential viral proteins involved in DNA packaging [40] and capsid assembly [41] respectively. UL87 promotes expression of late viral transcripts [42], and UL117 blocks cellular DNA synthesis [43]. Overall, the viral transcripts present at late time post infection may represent the minimum HCMV gene expression pattern compatible with both viral persistence and host cell survival.

## Supporting Information

S1 Fig **PCR product sequencing.** SYBR Green PCR products were isolated and purified from T98G AD169 and TR-infected samples 72 hrs p.i. and sequenced by ElimBio. Sequences were aligned with HCMV TR and AD169 genomes using the NCBI nucleotide alignment tool.
(PDF)

S2 Fig **Viral DNA quantification in long term infected glioma and GSC cells.** A–C. Purified plasmid DNA encoding for AD169 was used to construct standard curves of the indicated genes using TaqMan. Each sample was run in triplicate. D and E. 387 GSC (D) and T98G (E) cells infected with AD169 were used to harvest genomic DNA at indicated time points. 1ug of DNA was used to run Taqman for HCMV pp65, pp71 and US28 genes. Values were normalized to Rab14. Each sample was run in triplicate and copy numbers were estimated based on the standard curves shown in panels A–C.
(PDF)

S3 Fig **IE1 protein expression in 387 GSC**. Protein was extracted from uninfected 387 GSC and AD169-infected GSC at 9 and 11 wks p.i and probed for IE1 and actin.
(PDF)

S4 Fig **CMV gene expression in 3832 GSC sorted for CD133.** Primary-derived 3832 GSC cells were sorted into CD133+ and CD133− fractions using the Miltenyi AutoMACS system with CD133 microbeads. cDNA from each fraction was used to determine viral gene expression using a SYBR Green array with custom CMV primers. Heatmap represents ΔCt values of viral genes normalized to housekeeping gene RPL13A. Red shows higher gene expression and green shows lower gene expression.
(PDF)

S1 Table **Sequences for primers and TaqMan probes**. UL111A sequence was obtained from Chang WL, Baumgarth N, Yu D, Barry PA (2004) Human cytomegalovirus-encoded interleukin-10 homolog inhibits maturation of dendritic cells and alters their functionality. J Virol 78: 8720–8731.
(PDF)

S2 Table **List of primers used for SYBR Green RT-PCR.** All primer sequences are displayed for each viral gene tested.
(PDF)
