# Supplementary material for: Human Cytomegalovirus Gene Expression in Long-Term Infected Glioma Stem Cells
Source: PLoS One. 2014 Dec 30;9(12):e116178. doi: 10.1371/journal.pone.0116178 (PMC4280176; doi:10.1371/journal.pone.0116178)
Supplement: S1 Fig — PCR product sequencing. SYBR Green PCR products were isolated and purified from T98G AD169 and TR-infected samples 72 hrs p.i. and sequenced by ElimBio. Sequences were aligned with HCMV TR and AD169 genomes using the NCBI nucleotide alignment tool. (PDF) [file pone.0116178.s001.pdf]

UL87

```
UL87 TR PCR          TCGTCTGTTACTGGGACCCGTGGCCGTACCCTGTTTTTGCGACG
                     ||||||||||||||||||||||||||||||||||||||||||||
HMCV TR 129945       TCGTCTGTTACTGGGACCCGTGGCCGTACCCTGTTTTTGCGACG  129988
```

```
UL87 AD169 PCR       GTCTGTTACTGGGACCCGTGGCCGTACCCTGTTTTTGCGACG
                     ||||||||||||||||||||||||||||||||||||||||||
HCMV AD169 128620    GTCTGTTACTGGGACCCGTGGCCGTACCCTGTTTTTGCGACG  128661
```

UL115

```
UL115 TR PCR         ACAGGCATCGGCGCGTTAGTTCGGGGCACTCC
                     ||||||||||||||||||||||||||||||||
HCMV TR 165722       ACAGGCATCGGCGCGTTAGTTCGGGGCACTCC  165753
```

```
UL115 AD169 PCR      CTCACCCAACAGGCATCGACGCGTTAGTTCGGGGCACTCC
                     ||||||||||||||||||||||||||||||||||||||||
HCMV AD169 164357    CTCACCCAACAGGCATCGACGCGTTAGTTCGGGGCACTCC  164396
```

UL95

```
UL95 TR PCR          AGTCGGCCGAGGTGCCGGTGTTGGATAGCAGTAGCAGCGG
                     ||||||||||||||||||||||||||||||||||||||||
HCMV TR 140204       AGTCGGCCGAGGTGCCGGTGTTGGATAGCAGTAGCAGCGG  140243
```

```
UL95 AD169 PCR       GCGCGAATCGGCCGAGGTGCCGGTGTTGGATAGCAGTAGCAGCGG
                     |||||||||||||||||||||||||||||||||||||||||||||
HCMV AD169 138870    GCGCGAATCGGCCGAGGTGCCGGTGTTGGATAGCAGTAGCAGCGG  138914
```

UL55

```
UL55 TR PCR         ATGCTGTCGACGGTGGAGATACTGCTGAGGTC
                    ||||||||||||||||||||||||||||||||
HCMV TR 82900       ATGCTGTCGACGGTGGAGATACTGCTGAGGTC  82931
```

```
UL55 AD169 PCR      GATCATGCTGTCGACGGTGGAGATACTGCTGAGGTCAATCATGCGT
                    ||||||||||||||||||||||||||||||||||||||||||||||
HCMV AD169 81549    GATCATGCTGTCGACGGTGGAGATACTGCTGAGGTCAATCATGCGT  81594
```

IE1

```
IE1 TR PCR          CTGCATGAAGGTCTTTGCCCAGTACATTCTGGGGGCCGATCCTCTGAGAGTCTGCTC
                    |||||||||||||||||||||||||||||||||||||||||||||||||||||||||
HCMV TR 172786      CTGCATGAAGGTCTTTGCCCAGTACATTCTGGGGGCCGATCCTCTGAGAGTCTGCTC  172730
```

```
IE1 AD169 PCR       GAGGAGATCTGCATGAAGGTCTTTGCCCAGTACATTCTGGGGGCCGATCCTCTGAGAGTCTGCTC
                    |||||||||||||||||||||||||||||||||||||||||||||||||||||||||||||||||
HCMV AD169 172407 GAGGAGATCTGCATGAAGGTCTTTGCCCAGTACATTCTGGGGGCCGATCCTCTGAGAGTCTGCTC
```

S1 FIGURE
