# Supplementary figures and images for: Human Cytomegalovirus Gene Expression in Long-Term Infected Glioma Stem Cells
Source: PLoS One. 2014 Dec 30;9(12):e116178. doi: 10.1371/journal.pone.0116178 (PMC4280176; doi:10.1371/journal.pone.0116178)

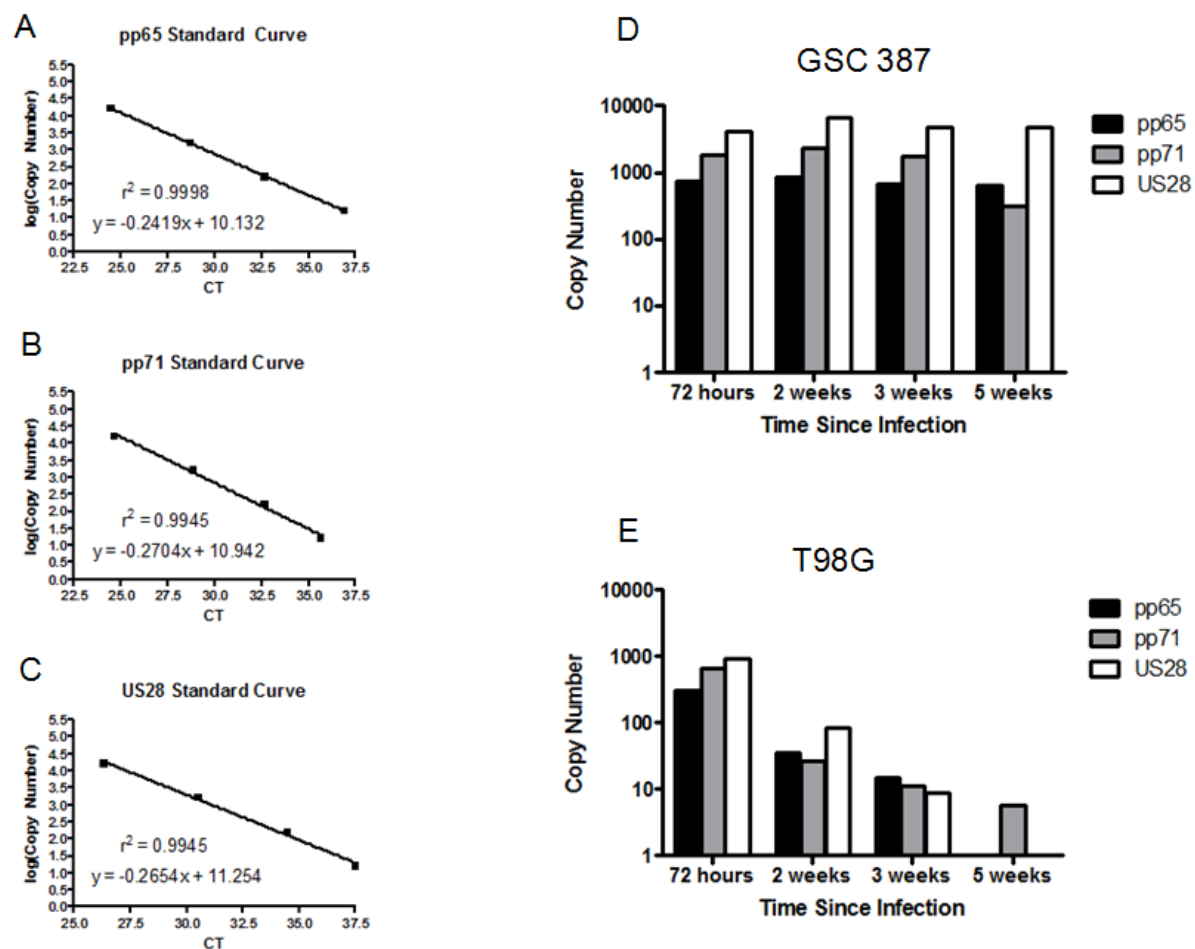

**S2 FIGURE**

Supplement: S2 Fig — Viral DNA quantification in long term infected glioma and GSC cells. A–C. Purified plasmid DNA encoding for AD169 was used to construct standard curves of the indicated genes using TaqMan. Each sample was run in triplicate. D and E. 387 GSC (D) and T98G (E) cells infected with AD169 were used to harvest genomic DNA at indicated time points. 1ug of DNA was used to run Taqman for HCMV pp65, pp71 and US28 genes. Values were normalized to Rab14. Each sample was run in triplicate and copy numbers were estimated based on the standard curves shown in panels A–C. (PDF) [file pone.0116178.s002.pdf]

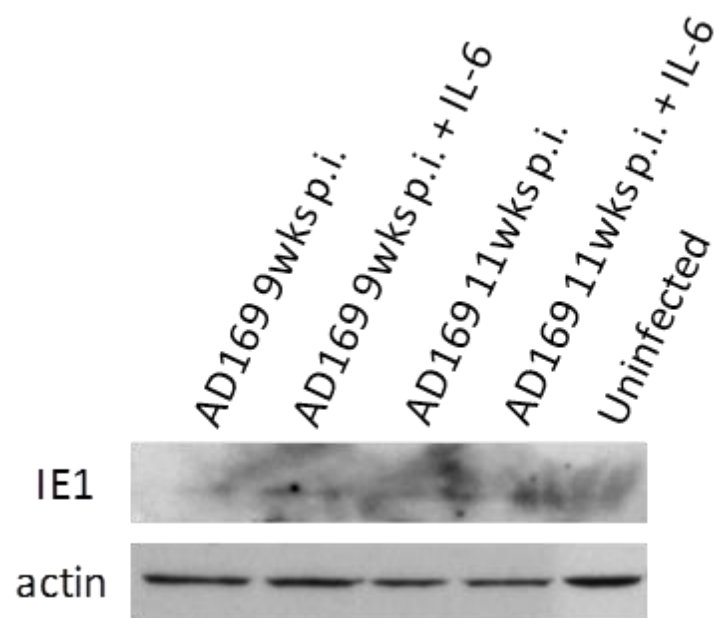

**S3 FIGURE**

Supplement: S3 Fig — IE1 protein expression in 387 GSC. Protein was extracted from uninfected 387 GSC and AD169-infected GSC at 9 and 11 wks p.i and probed for IE1 and actin. (PDF) [file pone.0116178.s003.pdf]

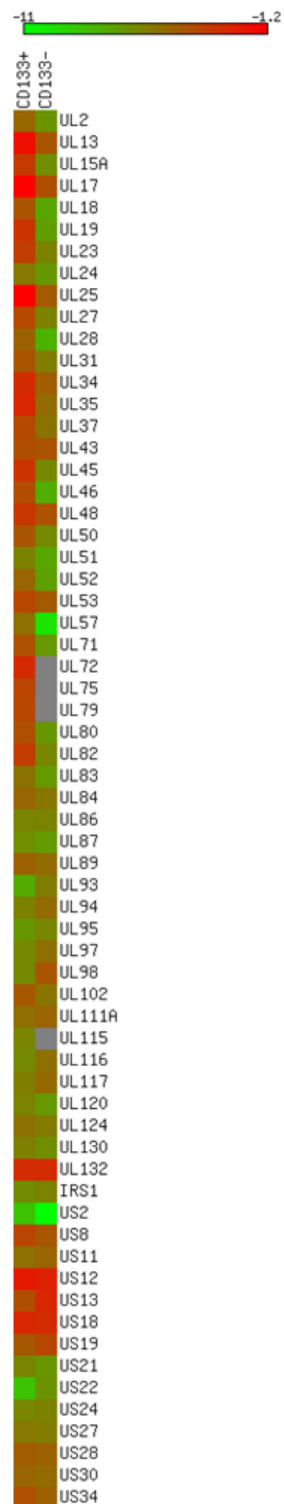

**S4 FIGURE**

Supplement: S4 Fig — CMV gene expression in 3832 GSC sorted for CD133. Primary-derived 3832 GSC cells were sorted into CD133+ and CD133− fractions using the Miltenyi AutoMACS system with CD133 microbeads. cDNA from each fraction was used to determine viral gene expression using a SYBR Green array with custom CMV primers. Heatmap represents ΔCt values of viral genes normalized to housekeeping gene RPL13A. Red shows higher gene expression and green shows lower gene expression. (PDF) [file pone.0116178.s004.pdf]
