# Supplementary material for: Human Cytomegalovirus Gene Expression in Long-Term Infected Glioma Stem Cells
Source: PLoS One. 2014 Dec 30;9(12):e116178. doi: 10.1371/journal.pone.0116178 (PMC4280176; doi:10.1371/journal.pone.0116178)
Supplement: S1 Table — Sequences for primers and TaqMan probes. UL111A sequence was obtained from Chang WL, Baumgarth N, Yu D, Barry PA (2004) Human cytomegalovirus-encoded interleukin-10 homolog inhibits maturation of dendritic cells and alters their functionality. J Virol 78: 8720–8731. (PDF) [file pone.0116178.s005.pdf]

S1 Table

Gene	Forward Primer	Reverse Primer	FAM/TAMRA Probe
IE1	AAGCGGCCTCTGATAACCAAG	GAGCAGACTCTCAGAGGATCG	CATGCAGATCTCCTCAATGCGGCG
US28	CGGCAACTTCTTGGTGATCTTC	CATCGCCGGAGCATTGA	CCATCACCTGGCGACGTCGGA
UL55 (gB)	AGGTCTTCAAGGAACTCAGCAAGA	CGGCAATCGGTTTGTTGTAAA	CCGTCAGCCATTCTCTCGGC
UL82 (pp71)	TCAGGCCGTTCATTTGGAA	AACCCACGGCGGAAAAAG	CCGACAGCCGCTAGGCCGC
UL83 (pp65)	GCAGCCACGGGATCGTACT	GGCTTTTACCTCACACGAGCATT	CGCGAGACCGTGGAACTGCG
UL111A	TGTTGAGGCGGTATCTGGAGA	CCGTCTTGAGTCCGGGATAG	CGTGTTTCCCGCAGGCGACC
