# Supplementary material for: Human Cytomegalovirus Gene Expression in Long-Term Infected Glioma Stem Cells
Source: PLoS One. 2014 Dec 30;9(12):e116178. doi: 10.1371/journal.pone.0116178 (PMC4280176; doi:10.1371/journal.pone.0116178)
Supplement: S2 Table — List of primers used for SYBR Green RT-PCR. All primer sequences are displayed for each viral gene tested. (PDF) [file pone.0116178.s006.pdf]

S2 Table

Gene	Forward Primer	Reverse Primer	Product Length
RL1	CCAACTTCACGCCGAAAC	CAGGGACGTTTATCCTTTGG	108
TRL14	AGTGGTATCAGTGGCGCTTA	TGGATCATATTTGGGAAGTTTG	91
UL1	AATTATGATCTACGTTTTGATCCAC	CACGCTATGCGATTTACGTT	90
UL2	CGCGATCCTTATAGTCGAAG	AATATGCCGCGCAGTAGTC	102
UL4	CGCTTGACGTTGCTAAATCT	AGGACACGGTCAGGTTGTAA	109
UL5	CGTACCGCAATCCATCTGT	GGTAGCGACGAGACGTTGAT	91
UL6	GTGATGGTGCTCGTTGATTT	CATTAACAAACCACGTTCTTCA	90
UL7	GCCTCGGGAAAACAACAC	CAACTTCCACCATCCCATAA	91
UL8	TTATCATCTGTCTGCGAGCAC	GCTCCGTGTCCGTCATAAAT	90
UL9	TCATCATTTACCTCAACCAACTT	TGTATGTGGGGTGGCAGTAT	93
UL10	TCAAGATTCCTCAAAGACTCAGA	TCACGATAAGTAGTCCGTAGTGG	95
UL11	CAAGCCCACAGTACCAACAC	ACAGCTTTTGCGGTATTCG	108
UL13	TGACGACAAATACGCTGACC	TCGAGACCCACAGTCCTAAG	90
UL14	GCTATCATCTACGGAGGGATT	ACACCAGTAGCACAGCAGGT	107
UL15A	AATGAAACGTCCGCCTGTA	AACCACGAACACAGTCCAAA	92
UL16	GGTCTCAGCCATCACTCTGT	GTGCGTAACCGCTGGTATC	108
UL17	CCGAGATTCCCAAAAGAAGA	CTCCCGAGGAAAGAAAAGTC	92
UL18	CCATCAGCAGTGTCCTCCTA	ACGCAAAGGCCAGGTTAC	105
UL19	GTGGATTGCGCCAGTTTAAT	GCGACTCACATCTAGCTCGTC	90
UL20	TGAAGTCCAGGTGCCTATCC	CTATTAAGTGGGGAGGTCGTG	110
UL21A	GTGCTACTGCTGGACGATGT	ACTGGTCCCAATGTTCTTCG	100
UL22A	CGGGGAGTATGACGTTTTG	CTTTGGCTTGATTTTCTTTGTG	92
UL23	ACAATCAGCACGTTTGCATC	TGCTCCTGGTTCCGTAATAA	102
UL24	TATACCGGGGCCGTCTAC	TTCAAACAGCAGGTTAAGTCC	105
UL25	AAGCGTTTATGGAAGCGAAC	TCGTCGCGTTGAGGATAGT	94
UL26	ACTCGCCAACCTTTTCAGTT	CAAAGTTCTCACGGCTGATCT	92
UL27	AACACGTCCACGATCTGAAG	CTGCGGTAGTCGGGAATC	103
UL28	CTTCTCGCCGTTGGACTC	AAAACACGTACTCGCTGACG	92
UL29	GCCGAGGACAAGACCTTC	CCAGCCGCACCAACTTAT	100
UL30	GTCAACAGCGTGCCTTTT	TTGTGTGTGTGCTCCGTAAA	106
UL31	ACTCGTACAACCACCACACG	ATGGGGAGACGTGAGAAAGT	110
UL32	CATGCTTTCGTCAGCCTCT	GCCCAAAGTAGGTTTCAAGG	106
GPCR	TTCAATTTTCCCAGCGGTA	GGTTATGATGCGATTGTGAGA	100
UL34	AGAACCCTCCGTCTCCAAC	GAGGTCCGTCTTCTTCGTC	98
UL35	ACCCTCTTTTCGCCCTACTC	GTCCCTGGAAGCGTGTGT	109
UL36	GGGGACTTTAACGGTCTCAA	ACGGAGCTGGATGAAGCA	93
UL37	CTCTCCCGCCTTGGTTAAG	CCGAGTTCTCACCGTCAAT	102
UL38	GTCGAGAGCGAGTTGTCCTC	GACCACGACCACCATCTGTA	105
UL40	CGCTCGACGTGTACTGTTG	CGCGTGTTGTTACCCACTT	106
UL41A	CTTTATTCTCATCTTTTGCTCCA	TTTTCCCAAACCGTGGTC	108
UL42	GCTGGAAATGGACTTTTGCT	CCCGTTGTTATATTGGCACT	109
UL43	GTTTTACCCACCCTGAAAGC	TCACCTTCGAGCAAAGAGC	110
UL44	CAACTCCGGCAATTACTTCA	GCACTTTTGCTTCTTGGTGT	97
UL45	GAGCAGCACCAGGAGGAG	ACAAACACGCTCAGGTTCAC	104
UL46	ATACAAAAGCTGGGCTGGTG	CGACGCTAAAGTTGTCCAAA	103

**UL47**	CCTTACGCGAGTTCTTGGAG	GCCGTTGATTCTTCGTGAT	94
**UL48**	ACATCAAGGACAGCCGACTC	ACGGCCACAAGTTCATCTC	110
**UL48A**	GAGCTGCCGACCGAGATA	GGCGCACTTGTCATTAAACA	99
**UL49**	CCCCGAAAAGGAAACGGTGA	GCTACGACACCGACTACCTG	120
**UL50**	AACGAGGGCGAGTACGAG	CTTACGGTGACGTGGTTCC	92
**UL51**	TGAACGCGGGTCAGAAGTA	GCCGTTAATAGTTGCGTCTTC	106
**UL52**	ACAAAACGCACCTACAAAGG	GGTCCACCAGCTTGATGTC	94
**UL53**	CAGCCCCACCACTACTCTC	GCACGAATGCTGTTGAGAAA	98
**UL54**	TACGAGGTAGCCGAAGATCC	GGCGACAGCACGTTAGTTAC	107
**UL55**	TCCAGTACCCTGAAGTCGGT	ACGCATGATTGACCTCAGCA	93
**UL56**	CGACACGCAAGAGACCAC	GCAGACTACCAGGCACCAG	90
**UL57**	TCCGACGATGTGCTCTTCT	TTCTCCAGCGAAAAGTCCTC	110
**UL69**	AATCACAGTACGACGGCAAC	AGCGCATTACCACCGAGT	94
**UL70**	AGCACAGCTACGCGCTCT	GCGGTCTGATTTTGATGGA	102
**UL71**	GAACGCGAGATTTTGGATCT	AAAGGGTATCTGGGGAGGAG	102
**UL72**	CGACGAGACAGAAGAAGATGAA	GGATGACGGGGAGGTTTC	99
**UL73**	GGCCTGGTGGACTATGCTT	CTTTGGTGGTGGTTGCAGTA	101
**UL74**	GTAACTTTAGCCTCCGGTCAC	TACTGCAACCACCACCAAAG	106
**UL75**	GCGAACTCACTACCTCATGC	ATAGCGCGTAGACGGACATC	110
**UL76**	TTTTACCGGGCTTTTCGTT	AAAGGTGTGCAACAGACTCATC	97
**UL77**	CACTATCACCCGGGCACTTT	CAGCTCCTCCGTTATCGTCC	105
**UL78**	GTCGGAGAAAGCCACGTT	GCAGAGGACGGAGGAACTT	103
**UL79**	ACACGAGACGGGCATTTT	GTGCTGCTGCTGTTGTGG	103
**UL80**	GCCTCTTCCTCCCCAACTAC	TCAGCTACCTTGGCACCTC	110
**UL82**	GCTCTCACGCTCGTCATC	CGCAGATAAGAGGTAGGTCGT	110
**UL82**	ACCTGGGGACGCTTGATTTT	GGCCGTTCATTTGGAACACC	108
**UL83**	GCTACGGTTCAGGGTCAGA	GCCATACGCCTTCCAATTC	94
**UL84**	GAGACGGAACCGCAAGAC	TTAGAGATCGCCGCAGAC	90
**UL85**	CACCTGCTGATCGACATGA	CTGTCCTCTAGCGCCAACTT	107
**UL86**	CGCTGATGGAGACCAAACT	CGGGATGATTTCGCCTAC	92
**UL87**	CGATACACGTGAGGACCGAG	CGTCGCAAAAACAGGGTACG	90
**UL88**	GCCTGTCTGTGTTTGTTGCT	ACAAGGTCGTTCTCCTCGAC	98
**UL89**	CGTGTCGCAGTTTGTGCT	ATCACGTTGTCCTTGTTCTCG	90
**UL91**	TGACGGTGGAGTGCGTTA	CGGAGGGAACAGGAGAAG	108
**UL92**	CGCGTGTGTCATCAAGGTTA	ATCCGAATACAGGTGCGTTT	92
**UL93**	CGAGACCGATTCCAGAACTT	CCGATACTCGCGGTGTTT	91
**UL94**	GGAGACGGCCTGGATTAT	CAGGTTCCTCTACCTTGACCA	100
**UL95**	CCCCATCGTTATCCAAGACT	ACTTGCCGACGTGGTAGAC	110
**UL95**	GCCTAATTGAAACGCCCGAC	CCGCTGCTACTGCTATCCAA	97
**UL96**	CTGCAATCCGGTAACGTG	GAAACCGAGTCCACCAAGG	95
**UL97**	AATACGTCAGAAAGAACGTGGA	GGTCCTCCTCGCAGATTATG	102
**UL98**	TGCCGTCTCTACCGTGCTA	GAACTCACAACATATTCGTTTGC	102
**UL99**	AAACCGAGCAAGCAGTCAA	GTCTAGGTCGTCCGTCTCCT	93
**UL100**	ATACGTCAAGGTGCAATTCG	GTGCGGTATTCGTTGCTG	108
**UL102**	CTGGGCGCGAGAGTTATT	CGAAGAGGACGAAGTGACG	103
**UL103**	GGCCTGCTAGACGTGCTC	TCACTCTTCCTCTCCTCGTTC	90
**UL104**	CGATACGTGGAGCTGATTTG	AGTTCCCCTTTCTGGCACT	90
**UL105**	GGACCCCGAACACCTCAT	GTGGTGCGTTTGTCTTTGAG	108

**UL111A**	GATTAGTCGGCTGTCTCAGGA	CGAGTGCAGATACTCTTCGAG	109
**UL112**	GTGGCGGTGGTAGAAACAG	GTCCTCCTCGTCCTCGTG	101
**UL114**	CATCTGGTGTTCATGCTGTG	GGGTGGCAGGACTTGAGTA	92
**UL115**	GTAACATTCACCAACGGGCG	GGAGTGCCCCGAACTAACG	93
**UL116**	TCACCTGCTGGATGCAATA	GCACGATGAGAGTGTGACG	98
**UL117**	TCGTAGAAAAGCAGCCAGAC	AGGCGAAAGCTGGTGAAT	109
**UL119**	GCCACCTGCAAAGTTATCCT	ATACCCCACCTGAGTGAAGG	108
**UL120**	CCGAGCCTTTATGATTGTCA	CACCTCTTGTGACGCCTAAA	102
**UL121**	GGAGAAAGAGTTGTGCATCG	TGAACGACAGGATCAGACAGA	110
**IE2**	GAGCCCGACTTTACCATCCA	CAGCCGGCGGTATCGA	
**IE1**	AAGCGGCCTCTGATAACCAAG	GAGCAGACTCTCAGAGGATCG	
**UL124**	CATCGTGCTGTGCCTAAGTC	CTACATCCGAGCCCTGCT	98
**UL128**	ATCCATGAGTCCCAAAGACC	AATTCGCAACATTCTTCTGC	102
**UL130**	CGGGACTACAGCGTGTCTT	CAAACGATGAGATTGGGATG	95
**UL132**	CTCGGAAATGGAAGAACCTT	CTAGTCGTACTCGGGATCTCTG	103
**RL13**	ACCACGACCGTTACTTGTGA	GCCACCAACGTATCCTGTTA	96
**IRS1**	CCAGGTCATCCCGTTGTG	GTCTGGCGGCTTATGTTTCT	103
**US1**	CGTGAAAAGACCCAAATTCC	GGACGTGCTGTCGTTCTC	100
**US2**	ATGGTGCGGAAGTCATACAC	CAGTCCACAGTCACATACACG	101
**US3**	TTCAGCGAAGCATGAGAGAC	CTACGGTCAGGAGCACAAGA	96
**US6**	GACACGGATTCTTCGCTGT	ATCCCACGTCCAGTCGAT	109
**US7**	CTGCATTGTCCGTTTTATAGG	GGTCAAAAGATTATATGTAGGTTTTCC	97
**US8**	GGTGCTGCTGTTGGGATAC	TTCACACTTTTGGGGTACACA	97
**US9**	GGTAGCCTGCTGTGTGTACC	TAGCCTCTTCTTCCCGTGAG	92
**US10**	GACCCCGAAACTACTCTGCT	AGTTCATCAAAACGCCCATC	108
**US11**	CTGATGATGGTGGCAGTGAT	ACTGGTCCGAAAACATCCAG	91
**US12**	GCACCGCTATGCTGCTAT	CCCGGCGTAGTTTGGATA	95
**US13**	GTTACATCCGCGACGAACT	GTTGGGACTGAGCACGAT	110
**US14**	TGGGACCAAATGTTCAGCTA	AGTGGTGGTGTTGAGGATGA	96
**US15**	CCGTCAAGGTGTTTCTCAGT	GCTTGTCAGAGGAAAAGTAGGTT	99
**US16**	GGTCCTTTTCGTCCGTCAC	CGGATTCGCAAGATCAGAA	105
**US17**	AGACAAACTCATCGCTTGGA	CTCTGTACCTCCCGCAAAAG	102
**US18**	TGTGTCAGCGTCGTGTTGTA	GTGTTTCTCGGCGTACTGCT	102
**US19**	CGCTGATGCTAATTCACGAT	TTTCGACATAGAGGCGTAGC	90
**US20**	ACGTTGTTGCTCATGCTCTC	TAGGACTTCCCCGTCGTACT	104
**US21**	TGCACGACATCGAGTATGAG	TCGGAGGGCATGAAGTAGA	100
**US22**	CGCCGACCACAGAGAAAG	GGATGGGGACCTGTTTCA	93
**US23**	GAAGCCGAAGTGGAAGCTC	TACACAAAGTGCTCCCGAAA	92
**US24**	GACACCAACTCGCAGAGCTA	GCCCGTATCGTCCTGATG	93
**US26**	AAGATAGACACGGCGCAAC	GGATGAGGCATGGTAGGTTT	98
**US27**	TGAGCTACAAAAGGACATACAAAGA	ATTCCTCCTCCCCGGACT	95
**US28**	GTACCACAGCATGAGCTTTTC	GTATAATTTGTGAGACGCGACA	101
**US29**	TCTCTGAAGGCCAAGACCAT	GAGGTGTCAACAACCCCAGT	91
**US30**	TGCAGGAAGAGAAGAAACGA	AGGAGCTGACGCTACAGGAG	110
**US31**	CCGTCTAACTGTGGGGAAAG	TAGTGTTGTTCACCCCGTGT	107
**US32**	ACGGCGAGAGGACGATCT	CACGTAAGTTTCCCGGTGAC	97
**US34**	AAAATGCTTGTCTGGCTGAG	GTACCGTTTTCCCATGACG	97

**US34A**	GTCGTCCAGTCGTTGTGC	GCATCACGCTCTTGTTTCAC	94
**TRSI**	TCTGCTTTTGTTCCATCTGTG	TGTGTGGCTATATTAGAAAACGTG	110
